# Inversion of the Vδ1 to Vδ2 γδ T cell ratio in CVID is not restored by IVIg and is associated with immune activation and exhaustion: Erratum

**DOI:** 10.1097/MD.0000000000006815

**Published:** 2017-04-28

In the article, “Inversion of the Vδ1 to Vδ2 γδ T cell ratio in CVID is not restored by IVIg and is associated with immune activation and exhaustion”,^[1]^ which appeared in Volume 95, Issue 30, the age ranges for CVID patients and for the healthy controls appeared incorrectly in the Study Cohort and Samples section. “Fifteen CVID patients (10 females and 5 males aged 6–51, average 34) from the Primary Immunodeficiency Outpatient Clinic of Clinical Immunology and Allergy Division of HCFMUSP, fulfilling the PAGID/ESID criteria (1999) for CVID diagnosis and 22 healthy controls (14 females, 8 males aged 25–45, average 36) were enrolled in the study” should have appeared as “Fifteen CVID patients (10 females and 5 males aged 6–62, average 34) from the Primary Immunodeficiency Outpatient Clinic of Clinical Immunology and Allergy Division of HCFMUSP, fulfilling the PAGID/ESID criteria (1999) for CVID diagnosis and 22 healthy controls (14 females, 8 males aged 17–64, average 36) were enrolled in the study.”
